# Development of a Fluorescence-based *Trypanosoma cruzi* CYP51 Inhibition Assay for Effective Compound Triaging in Drug Discovery Programmes for Chagas Disease

**DOI:** 10.1371/journal.pntd.0004014

**Published:** 2015-09-22

**Authors:** Jennifer Riley, Stephen Brand, Michael Voice, Ivan Caballero, David Calvo, Kevin D. Read

**Affiliations:** 1 Drug Discovery Unit, Division of Biological Chemistry and Drug Discovery, College of Life Sciences, University of Dundee, Dundee, United Kingdom; 2 CYPEX, Dundee, United Kingdom; 3 Molecular Discovery Research-Tres Cantos, GlaxoSmithKline, Centro de Investigación Básica, Tres Cantos, Spain; Northeastern University, UNITED STATES

## Abstract

Chagas disease, caused by the protozoan parasite *Trypanosoma cruzi (T*. *cruzi)*, is a life threatening global health problem with only two drugs available for treatment (benznidazole and nifurtimox), both having variable efficacy in the chronic stage of the disease and high rates of adverse drug reactions. Inhibitors of sterol 14α-demethylase (CYP51) have proven effective against *T*. *cruzi in vitro* and *in vivo* in animal models of Chagas disease. Consequently two azole inhibitors of CYP51 (posaconazole and ravuconazole) have recently entered clinical development by the Drugs for Neglected Diseases initiative. Further new drug treatments for this disease are however still urgently required, particularly having a different mode of action to CYP51 in order to balance the overall risk in the drug discovery portfolio. This need has now been further strengthened by the very recent reports of treatment failure in the clinic for both posaconazole and ravuconazole. To this end and to prevent enrichment of drug candidates against a single target, there is a clear need for a robust high throughput assay for CYP51 inhibition in order to evaluate compounds active against *T*. *cruzi* arising from phenotypic screens. A high throughput fluorescence based functional assay using recombinantly expressed *T*. *cruzi* CYP51 (Tulahuen strain) is presented here that meets this requirement. This assay has proved valuable in prioritising medicinal chemistry resource on only those *T*. *cruzi* active series arising from a phenotypic screening campaign where it is clear that the predominant mode of action is likely not via inhibition of CYP51.

## Author Summary

Chagas disease, caused by the parasite *Trypanosoma cruzi* (*T*. *cruzi*), is endemic in Latin America and emerging in North America and Europe through human migration. It is a severe global health problem with 8–10 million people infected and an estimated 12,000 deaths annually. Current treatment options are poorly efficacious and have severe side effects. New drugs are therefore urgently required. Two of these potential new drugs, posaconazole and ravuconazole, both targeting an enzyme in *T*. *cruzi* called CYP51, have recently failed in clinical development. Therefore, in light of these recent clinical failures and in order to better balance the overall risk in the drug discovery portfolio for Chagas disease, it has become prudent to assess whether new chemical start points for drug discovery programmes have a mode of action predominantly driven by *T*. *cruzi* CYP51 inhibition. In this paper we report a fluorescence based assay to determine whether compounds inhibit *T*. *cruzi* CYP51. This provides a high throughput screen to help prioritise medicinal chemistry resource on those *T*. *cruzi* active new chemical series that do not have a mode of action predominantly driven by CYP51 inhibition.

## Introduction

Chagas disease is a tropical parasitic disease caused by the flagellate eukaryotic (protozoan) parasite *Trypanosoma cruzi (T*. *cruzi)*, endemic in Latin America and now emerging in North America and Europe through human migration. It is becoming a severe global health problem with approximately 8–10 million people infected, an estimated 12,000 deaths per year, and placing 100 million people at risk. Transmission to humans and other mammals is predominantly by an insect vector, the blood-sucking "kissing bugs" of the subfamily Triatominae (family Reduviidae) [1]. Transmission has also been reported to occur through contaminated food, blood transfusions and from mother to child.

Clinical Chagas disease can be classified into two distinct phases, acute and chronic. In the acute phase, lasting a few weeks, parasites begin to multiply in the organs and tissues. Symptoms are usually mild and non-specific with patients rarely being diagnosed. However, life-threatening myocarditis or meningoencephalitis can occur during the acute phase with a death rate for people in this phase of about ten percent. Ten to fifty percent of infected survivors develop chronic Chagas disease. People in the chronic phase can be asymptomatic for many years, with parasites generally undetectable in the blood. However, the disease causes organ and tissue damage, particularly potentially lethal cardiopathy and megacolon or megaoesophagus, caused by the sequential induction of inflammatory response to the parasite. Nitroheterocyclic compounds, benznidazole and nifurtimox, developed in the 1960’s [2], are currently the only two drugs used for the treatment of Chagas disease. Both have low efficacy in the chronic stage and, with prolonged dosing regimens, both drugs have significant side effects including skin irritation, neurotoxicity, and digestive system disorders [3]. Newer, safer and more efficacious treatments are therefore in desperate need.

Inhibition of sterol 14α-demethylase (CYP51) has been considered a viable target against *T*. *cruzi* for over 30 years [2,4,5,6,7,8]. Found in a broad variety of organisms including animals, plants, fungi and protozoa, this enzyme plays an essential role in the sterol biosynthetic pathway, catalysing the oxidative removal of the 14α-methyl group from sterol precursors such as lanosterol or eburicol [9]. The products of the pathway, cholesterol in humans or ergosterol in fungi, are required for the integrity of the eukaryotic cell membrane. These sterols are required for membrane function in *T*. *cruzi*. Inhibition of CYP51 activity is lethal as the *T*. *cruzi* parasites are unable to scavenge and utilise host cholesterol [10]. The CYP51 gene is known to be expressed in all stages of the parasite life cycle and indeed it has also been shown to be up-regulated in multiplying forms [9]. As with other members of the Cytochrome family, CYP51 is a haem containing protein located on the membrane of the endoplasmic reticulum that relies upon electron transfer by NADPH reductase for activation [11].

**Fig 1:**
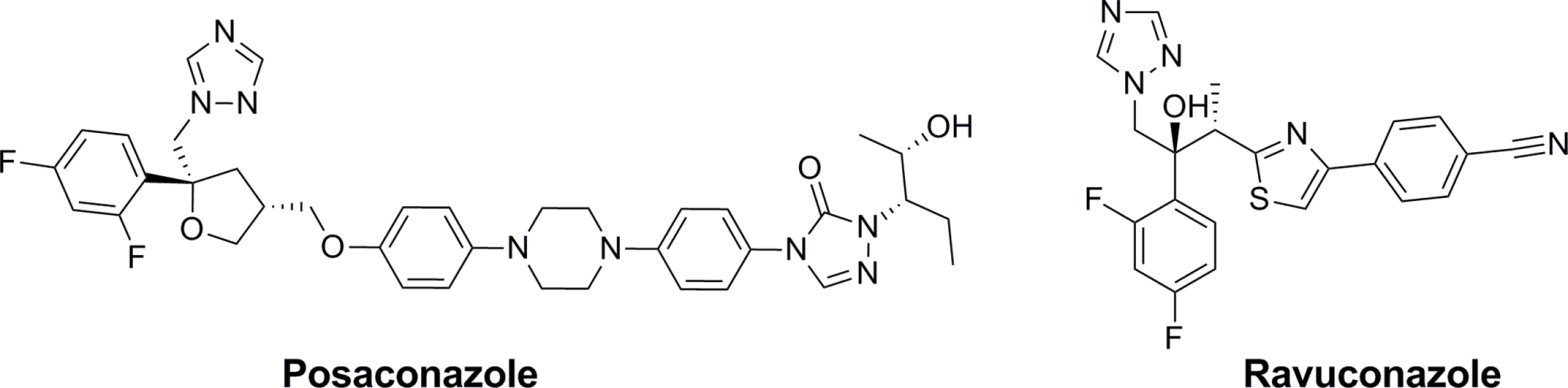
Clinical candidates for Chagas disease.

Azole inhibitors, which interfere with sterol biosynthesis, essential in eukaryotic cells, have already been used with success in humans in the treatment of fungal infections. Several of these drugs have been considered as possible treatments for Chagas disease [12]. Ketoconazole, fluconazole, itraconazole, ravuconazole and posaconazole are known to inhibit CYP51 *in vitro*, competitively binding to the haem within CYP51 and occupying the active site preventing any substrate from binding. Although ketoconazole and itraconazole have not demonstrated significant curative activity in humans with chronic Chagas disease [6], other azoles, with greater potency and improved pharmacokinetic properties, which have been shown to have potent activity against *T*. *cruzi*, including posaconazole [13,14] and ravuconazole (Fig 1), are in clinical development with the Drugs for Neglected Diseases initiative (DNDi).

To prevent enrichment of candidates against a single target, and thus reduce risk in the overall drug discovery portfolio for Chagas disease, it has therefore become necessary to evaluate and prioritise medicinal chemistry resource on new chemical series active against *T*. *cruzi* but with such activity not likely driven via *T*. *cruzi* CYP51 inhibition. Recent findings from clinical trials with posaconazole [15] and ravuconazole [16] has indicated re-emergence of parasitaemia in two thirds of patients once dosing has been completed, thus reinforcing the need to strengthen the overall drug discovery portfolio for Chagas disease with new chemical lead series not working via this mechanism of action.

Evaluating compounds as potential inhibitors of *T*. *cruzi* CYP51 has previously been demonstrated measuring the apparent dissociation constants (Kd) by spectral titration [4,17,18] utilising the shift of the haem iron soret band in response to binding [18]. One of the drawbacks to this methodology is that micromolar protein concentrations are required for screening causing potential interference with the optical properties and/or solubility of test compounds [4]. There are many potential reasons why affinity estimates measured by binding may not correlate with functional inhibition. These include allosteric sites, non or uncompetitive modes of inhibition or slow kinetics [19].

**Fig 2:**
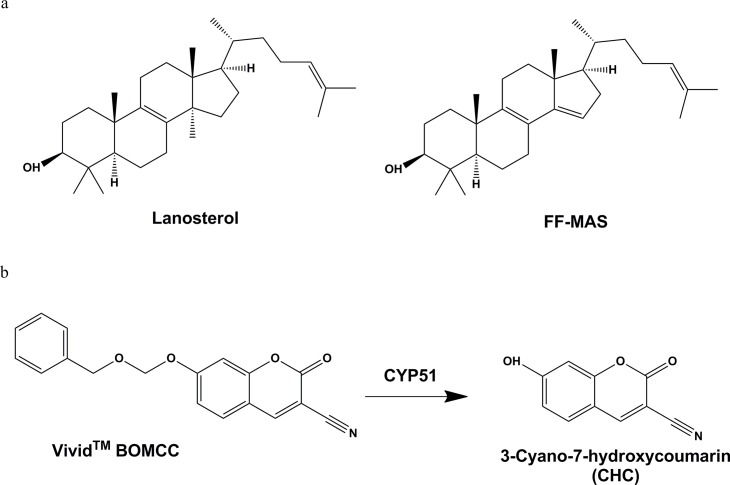
a: Traditional endogenous substrate (Lanosterol) and CYP51 specific metabolite (FF-MAS); b: Fluorogenic probe substrate BOMCC is metabolised to CHC by CYP51.

Inhibition of endogenous substrate lanosterol, eburicol and obtusifoliol has also been used as an *in vitro* tool using recombinant expressed human CYP51 enzyme [20]. In particular, measuring effect on CYP51 driven metabolism of lanosterol to follicular fluid meiosis activating sterol (FF-MAS) in the presence of test substances [21] is well established (Fig 2). However, FF-MAS detection requires mass spectrometry limiting the number of compounds that can be tested and consequently limiting the value of such an assay for triaging large numbers of phenotypic screening *T*. *cruzi* hits toward identifying modes of action away from CYP51.

Metabolism of fluorogenic probe substrate to a product, detectable by fluorescence is well established with recombinantly expressed cytochrome P450 enzymes (CYP’s) for the purpose of assessing possible drug-drug interactions [22]. Measuring CYP inhibition by this method provides a high throughput screening approach, avoiding time consuming analysis by mass spectrometry and minimising use of expensive substrates. The O-dealkylation of Vivid substrate benzyloxymethylocyanocoumarin (BOMCC) to fluorescent product cyanohydroxycoumarin (CHC) is commonly used to evaluate CYP3A4 activity in recombinantly expressed membrane preparations (Fig 2). Valuably, O-dealkylation activity in the presence of recombinantly expressed *T*. *cruzi* CYP51 was observed. This has enabled the creation of a fast, high-throughput, 96 and 384 well microtitre method to assess the inhibitory potential of compounds against *T*. *cruzi* CYP51, which is described in this paper.

## Materials and Methods

### Chemicals and reagents

Benzyloxymethyloxycyanocoumarin (BOMCC) was obtained from ThermoFisher Scientific. Fluconazole, ketoconazole, itraconazole, NADP, NADPH, glucose-6-phosphate, glucose-6-phosphate dehydrogenase, Cytochrome C from horse heart and sodium bicarbonate were obtained from Sigma Aldrich. 50 mM potassium phosphate (pH 7.4) buffer was prepared from dibasic and monobasic forms of potassium phosphate obtained from Sigma Aldrich.

### Cloning and expression of *T*. *cruzi* CYP51 in *E*. coli

*E*. *coli* membrane fractions containing the *T*. *cruzi* CYP51 (Tulahuen strain) (Bactosomes) were provided by Cypex Ltd.

The cDNAs coding for *T*. *cruzi* CYP51 and NADPH P450 reductase were synthesized and supplied in pUC vectors by Genescript. The CYP51 cDNA was cloned into the expression vector pCW with an *ompA* N terminal leader and the reductase was cloned into a pACYC184 derived expression vector with a *pelB* N terminal leader. *E*. *coli* JM109 was used for the co-expression of the recombinant proteins.

### Data analysis

For each concentration of test compound the rate of fluorescence units per minute was calculated as a percentage of the average rate of the solvent only control wells.

The percentage of solvent control values was then plotted against the concentration range. Using the following 4 parameter fit equation, an IC_50_ value can be determined.

$$y=\frac{Range}{1+{\left(\frac{x}{IC_{50}}\right)}^{s}}+Background$$

### Accession numbers

Accession number for *T*. cruzi cDNA is AY283022; *T*. cruzi reductase is DQ857724 and mosquito reductase is AY183375.

## Results and Discussion

### *T*. *cruzi* CYP51 reductase partner

Despite the presence of the N terminal *pelB* leader sequence, the recombinant *T*. *cruzi* NADPH P450 reductase proved to be a cytosolic protein (as might be expected from previous data [10]) and was not present in the *E*.*coli* membrane fraction with the *T*. *cruzi* CYP51. Reconstitution of the cytosolic fraction containing the *T*. *cruzi* reductase with the membrane fraction containing the *T*. *cruzi* CYP51 did not result in active CYP51 so, in order to generate an active *T*. *cruzi* CYP51 system, the *T*. *cruzi* CYP51 was co-expressed with human (expression construct supplied by Cypex Ltd) or mosquito (expression construct supplied by the Liverpool School of Tropical Medicine) NADPH P450 reductase. Screening of CYP51 activity showed that the *T*. *cruzi* CYP51 co-expressed with the mosquito reductase gave the highest rate of BOMCC turnover. The mosquito reductase expression cassette was cloned from the pACYC vector into the pCW based CYP51 expression vector to allow both proteins to be expressed from the same plasmid resulting in a relatively higher level of mosquito reductase in the membrane fraction with a concomitant increase in BOMCC turnover. Supplementation of the *T*. *cruzi* CYP51 / mosquito NADPH P450 reductase bactosomes with partially purified mosquito cytochrome *b*_5_ at 10 fold excess over the CYP51 resulted in a further increase in BOMCC turnover. This bactosome preparation was then scaled up for all further work.

### Determination of kinetic parameters

**Fig 3:**
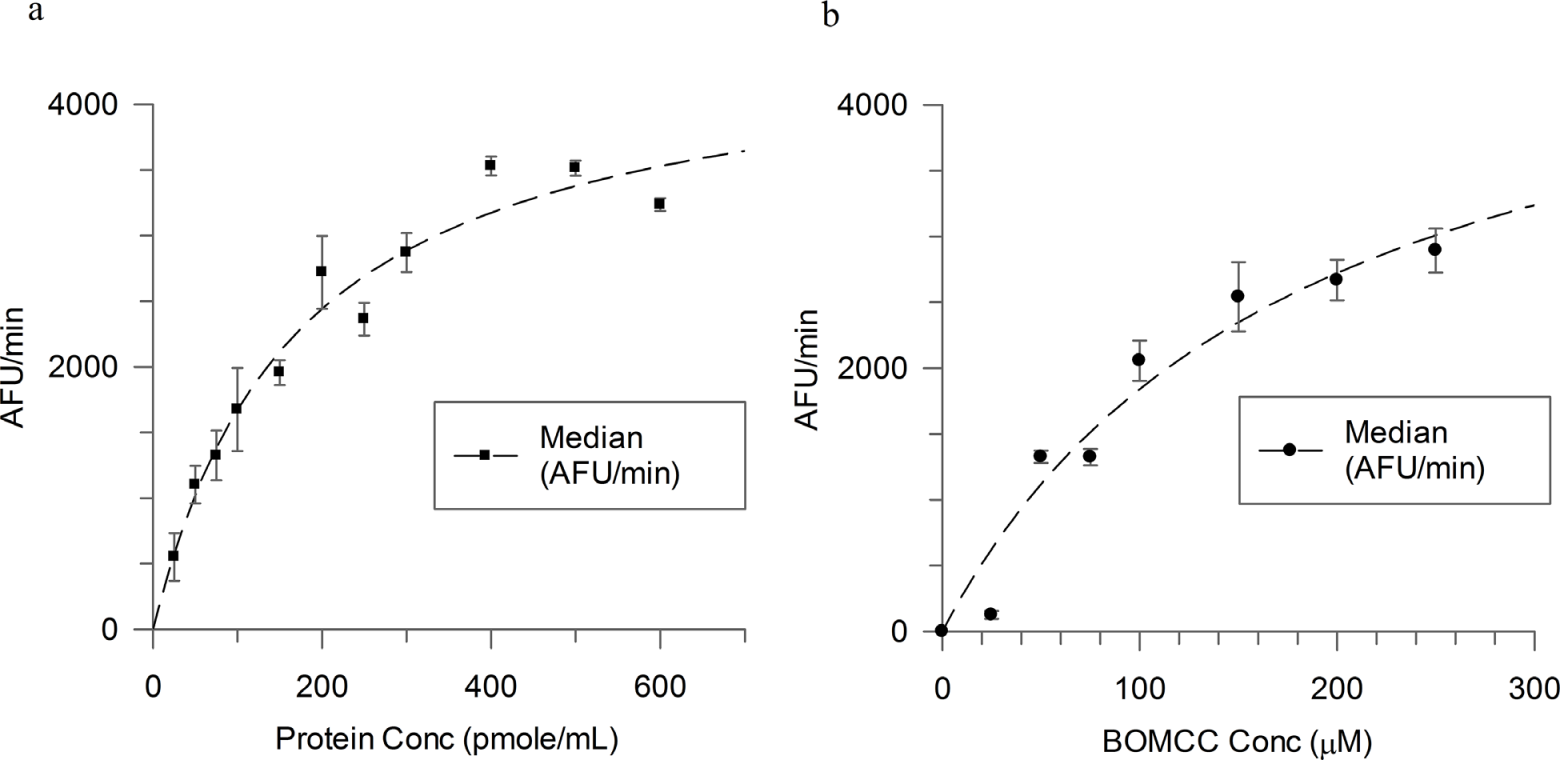
a: Linearity of CHC production (expressed as average rate of fluorescence units (AFU)) with increasing T. cruzi CYP51 enzyme concentration (96 well format; average points (n = 3) with error bars); b: Michaelis-Menten Kinetic determination (96 well format; average points (n = 3) with error bars).

To determine kinetic parameters, 100 μM BOMCC O-dealkylase activity was assessed in incubations containing 25, 50, 75, 100, 150, 200, 250, 300, 400, 500 and 600 pmoles/mL *T*. *cruzi* CYP51 enzyme. Protein concentrations were normalised using control protein containing no CYP activity. Incubations were pre-warmed at 37°C before addition of NADPH regenerating system. Production of CHC was measured at 1 minute intervals at 37°C (Exc 410 nm, Em 460 nm) for 10 minutes (Fig 3a).

Km and Vmax parameters for BOMCC were determined by incubating 37 pmoles/mL *T*. *cruzi* CYP51 enzyme (0.24 mg/mL bactosomes) with 25, 50, 75, 100, 150, 200 and 250 μM BOMCC. Incubations were pre-warmed at 37°C before addition of NADPH regenerating system. Production of CHC was measured at 1 minute intervals at 37°C (Exc 410 nm, Em 460 nm) for 10 minutes (Fig 3b). Rates of BOMCC metabolism appeared linear up to 100 pmoles/mL with a Km value determined as 191± 55 μM. Assay conditions of 37 pmoles/mL of *T*. *cruzi* CYP51 in bactosomes and 100 μM BOMCC provided a good dynamic range of metabolism.

**Fig 4:**
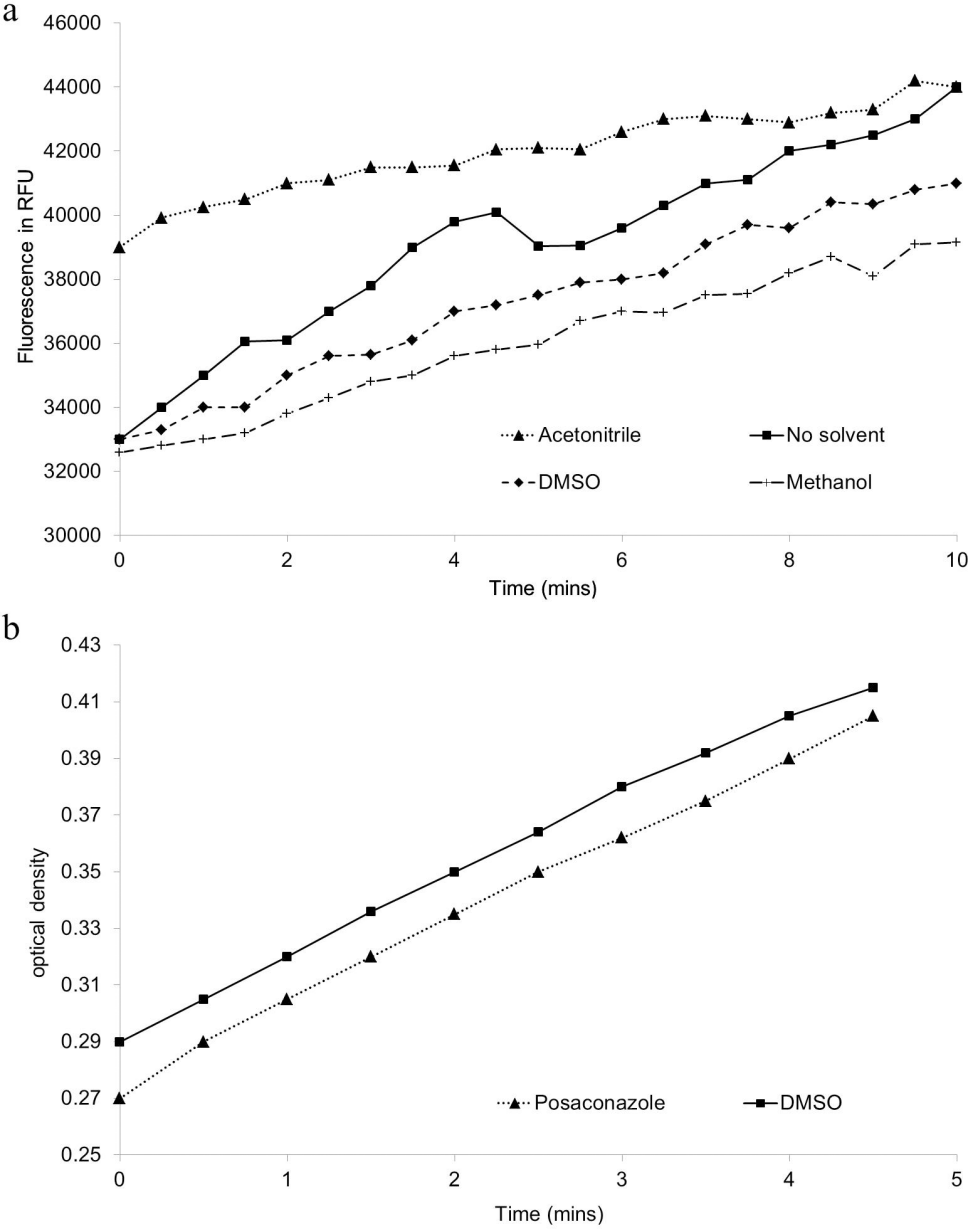
a: The effect of solvent on *T*. *cruzi* CYP51 activity; b: Cytochrome C reductase activity of recombinant preparations.

Metabolic activation of 100 μM BOMCC by *T*. *cruzi* CYP51 (37 pmoles/mL) was evaluated in the presence of 2% v/v DMSO, methanol or acetonitrile. Incubations were pre-warmed at 37°C before addition of NADPH regenerating system. Production of CHC was measured at 1 minute intervals at 37°C (Exc 410 nm, Em 460 nm) for 10 minutes. The average rate of fluorescence units (AFU) per minute was compared to a solvent only control (Fig 4a). There did not appear to be a significant decrease in metabolic activity in the presence of 2% v/v DMSO or 2% v/v methanol.

### Implementation of microtitre-based assay for compound screening

With kinetic parameters established, a microtitre-based assay to measure inhibition of recombinantly expressed *T*. *cruzi* CYP51 by test compounds was then implemented. Using a selection of ‘azole’ inhibitors, including those previously shown to competitively bind to CYP51, and known xenobiotic CYP450 inhibitors (Table 1), the assay was validated to ensure robustness. The incubation times and protein concentrations employed were within the linear range for each assay. Incubations containing 37 pmoles/mL of *T*. *cruzi* CYP51 in bactosomes, 100 μM BOMCC, 10, 3.3, 1.0, 0.33, 0.1, 0.033 or 0.01 μM test compound (2% v/v solvent) in 50 mM potassium phosphate buffer (pH 7.4), were pre-incubated at 37°C for 5 minutes. Upon addition of NADPH regenerating system (7.8 mg [28 μM] glucose-6-phosphate, 1.7 mg [2.2 μM] NADP, 6 units/mL glucose-6-phosphate dehydrogenase in 2% w/v sodium bicarbonate buffer) production of fluorescent metabolite CHC was measured (Exc 410 nm, Em 460 nm) at 1 minute intervals over a 10 minute period. Rates of metabolite production per minute were compared to uninhibited controls and plotted against test compound concentration to obtain an IC_50_. The assay was then miniaturized to 20 μL and moved from kinetic to single endpoint stopped readout (60 minute reaction before quenching with posaconazole to generate a long stable signal) to increase throughput.

**Table 1:** IC_50_ Determination of a test set of compounds.

Test Compound	Structure	IC_50_ (μM), n = 3	Reference, Kd (μM)
Ketoconazole		0.014^a^	0.2 [18]
Miconazole		0.057^a^	
Itraconazole		0.029^a^	
Posaconazole		0.048^a^	0.06 [24], 0.018 [20]
Fluconazole		0.880^a^	0.23 [18], 0.43 [24], 0.15 [25]
Methimazole		>8	
Ticlopidine		>8	
Sulphaphenazole		>8	
Sulphamethoxazole		>8	
Quinidine		>8	
Furafylline		>8	

^**a**^ Coefficient of variation for all IC_50_’s were lower than 1%

Ketoconazole, itraconazole, posaconazole and miconazole all showed potent inhibition of *T*. *cruzi* CYP51 activation of substrate BOMCC with IC_50_ values of 0.014, 0.029, 0.048 and 0.057 μM, respectively. Indeed, it is likely that these compounds are even more potent than they appear to be as the *T*. *cruzi* CYP51 enzyme concentration used in the assay defines a minimum IC_50_ of approximately 0.02 μM. Fluconazole also inhibited activity (IC_50_ 0.88 μM) although this was at least 20 fold less inhibitory than observed with other azole type compounds. Methimazole did not appear to inhibit activity (IC_50_ > 8 μM), perhaps as a result of an inability to adequately fill the active site and prevent substrate binding.

Compounds which did not appear to inhibit *T*. *cruzi* CYP51 in this assay included known CYP450 inhibitors ticlopidine (CYP2C19), sulphaphenazole (CYP2C9), sulphamethoxazole (CYP2C8/9), quinidine (CYP2D6) and furafylline (CYP1A2). This was expected as, unlike other drug metabolising CYP450s, CYP51 has a very narrow substrate specificity, being limited to endogenous sterols including eburicol and lanosterol [23].

### Assessment of cytochrome c reductase activity in presence of test compounds

As previously discussed, addition of an equivalent reductase enzyme (mosquito, *A*. *gambiae*) was required to deliver metabolic activity of *T*. *cruzi* CYP51. Due to the artificial nature of this pairing it was therefore necessary to confirm that any potent inhibition of CHC production was not the result of indirect inhibition of the electron transfer by NADPH Reductase. Thus, cytochrome c reductase activity of the bactosome preparation was monitored in the presence of all test inhibitor compounds individually. Incubations containing 0.82 pmoles/mL of *T*. *cruzi* CYP51 in bactosomes, 50 μM cytochrome c, 10, 3.3, 1.0, 0.33, 0.1, 0.033 or 0.01 μM test compound (2% solvent) in 50mM potassium phosphate buffer (pH 7.4), were pre-incubated at 37°C for 5 minutes. Absorbance at 550 nm was then measured for 3 minutes to ascertain a background level. After addition of NADPH (final concentration 80 μg/mL), absorbance was further measured over 5 minutes. The rate of reduction of cytochrome c at each test compound concentration was compared to an uninhibited control (Fig 4b). The assay was then miniaturized to 50 μL and moved from kinetic to single endpoint readout (60 minutes reaction) to increase throughput. No decrease in activity of cytochrome c reductase was observed for any of the test inhibitors. Furthermore, subsequent evaluation of a much larger set of *T*. *cruzi* CYP51 inhibitors has since been carried out and none have yet decreased cytochrome c reductase activity confirming that inhibition of BOMCC O-dealkylation observed is by direct inhibition of *T*. *cruzi* CYP51 and not by its effect on the cytochrome c reductase nor the regeneration system. This strongly suggests that the artificial nature of the pairing of *T*. *cruzi* CYP51 with *A*. *gambiae* NADPH reductase in the bactosomes used for this assay will not deliver false positives.

### Implementation in Chagas disease drug discovery screening cascade

Identifying the fluorogenic probe BOMCC has enabled development of a fluorescence based high throughput functional assay to determine *T*. *cruzi* CYP51 inhibition, suitable for compound triaging of *T*. *cruzi* phenotypic screening hits. This assay is now embedded in our Chagas disease screening cascade to prioritise compound series for progression into hit to lead and lead optimisation.

**Fig 5:**
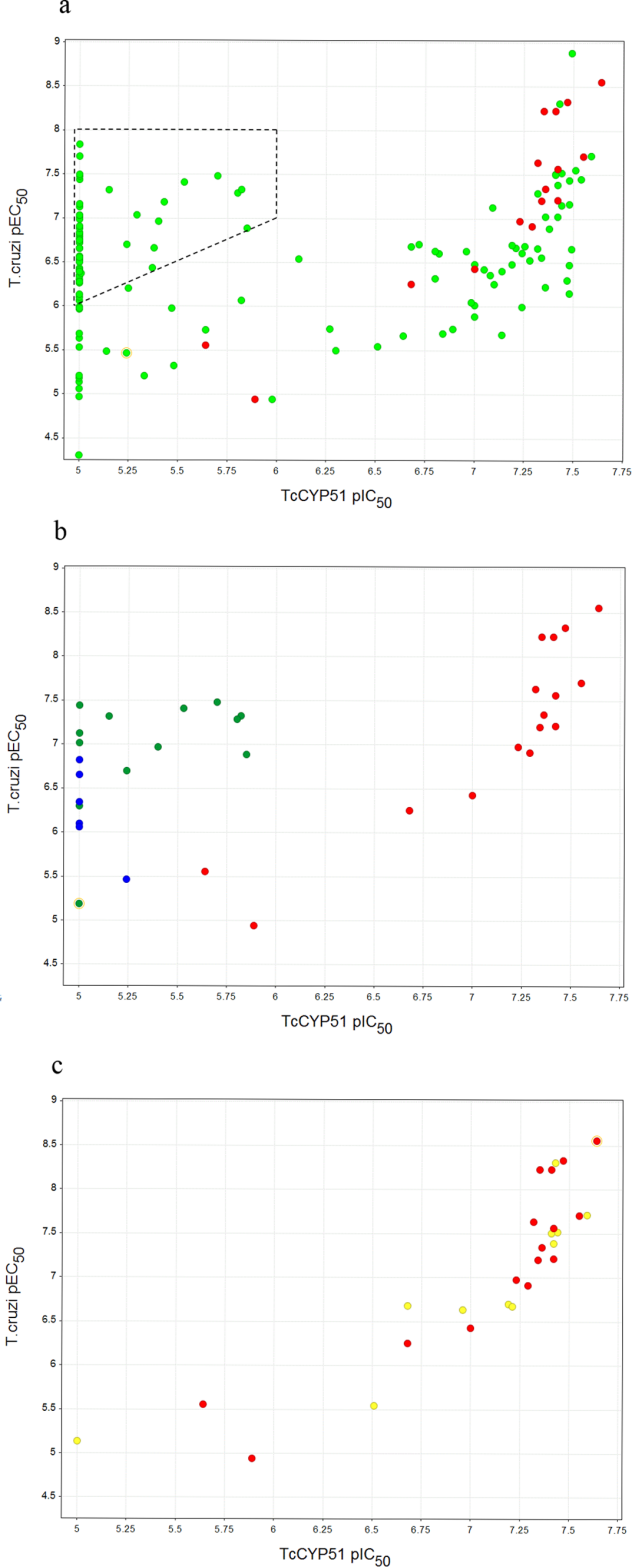
a: *T*. *cruzi* CYP51 inhibition plotted against *T*. *cruzi* activity for compounds from the different chemical series (coloured green), in addition to 16 known inhibitors of human CYP51 used as a reference set to define the correlation curve (coloured red). *T*. *cruzi* active series within the dotted box are prioritised for further medicinal chemistry; b: *T*. *cruzi* CYP51 inhibition plotted against *T*. *cruzi* activity for series one (green), and series two (blue) compared with the human CYP51 training set (red); c: *T*. *cruzi* CYP51 inhibition plotted against *T*. *cruzi* activity for a third series (yellow) compared with the human CYP51 training set (red). pEC_50_ or pIC_50_ is the −log of EC_50_ or IC_50_ respectively in M.

Following a phenotypic screen of approximately 200,000 compounds against the *T*. *cruzi* parasite, inhibition of *T*. *cruzi* CYP51 was measured for compounds from 22 trypanocidal series and 16 singleton compounds. Compounds were derived from a variety of sources including diversity and target-centric libraries from the Dundee Drug Discovery Unit and GSK corporate collections. At least three compounds from each chemical series displaying as wide a range of *T*. *cruzi* activity as possible were tested (a total of 129 compounds; Fig 5a).

With the assumption that those series which show a good correlation between T. cruzi activity and T. cruzi CYP51 enzyme inhibition have a T. cruzi CYP51 mediated mode of action, a remarkable enrichment for the *T*. *cruzi* CYP51 mode of action was observed. Correlation between *T*. *cruzi* activity and *T*. *cruzi* CYP51 inhibition was evident for 11 out of the 22 hit series, with an additional two series showing sporadic association with *T*. *cruzi* CYP51 inhibition. Similarly high rates were observed with the singletons, with 11 out of 16 showing antiparasitic activity in line with *T*. *cruzi* CYP51 inhibition. 13/22 series and 11/16 singletons were therefore de-prioritised from further medicinal chemistry resourcing and focus given to those hits demonstrating at least one log unit divergence between *T*. *cruzi* activity and *T*. *cruzi* CYP51 activity, providing confidence that the predominant mode of action was not *T*. *cruzi* CYP51 driven. It is clearly important to look at the overall correlation between inhibition of *T*. *cruzi* CYP51 and antiparasitic activity for a given series to ensure that they are not tracking.

**Fig 6:**
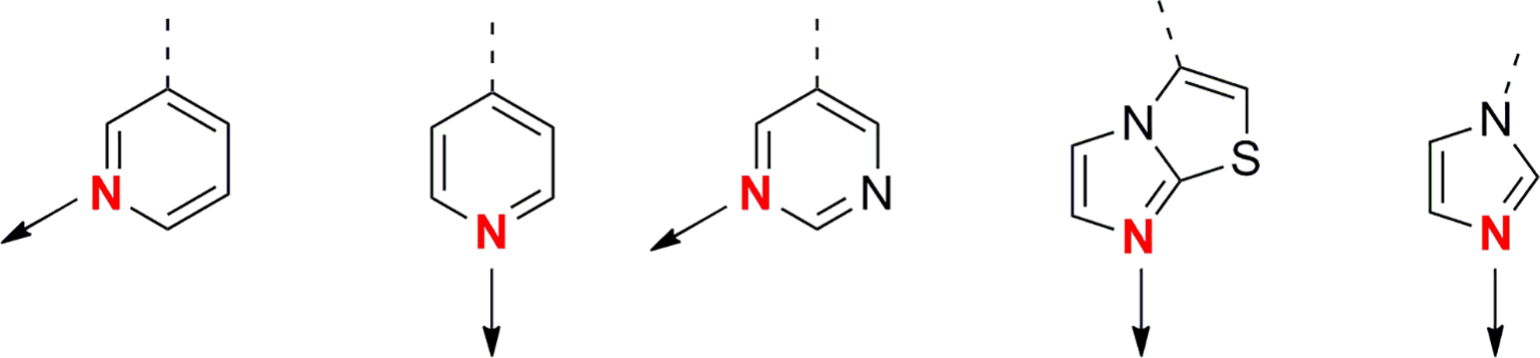
Chemical moieties associated with CYP inhibition.

As it transpires, the majority of the chemical series removed from progression bear aza-heterocyclic groups which are already well known to exert inhibition of cytochromes through co-ordination to the haem iron (Fig 6) [9] and could have perhaps been eliminated “by- eye”. However, the great benefit of this assay to our drug discovery effort is that it has allowed identification of *T*. *cruzi* active series which do contain potential cytochrome binding motifs, but which do not inhibit *T*. *cruzi* CYP51. For instance, two particularly promising series (Fig 5b) having potent *T*. *cruzi* activity were found not to have an obvious correlation with *T*. *cruzi* CYP51 inhibition in spite of containing heterocycles with known potential for haem-binding in cytochromes. This was in stark contrast to a third series (Fig 5c) where *T*. *cruzi* activity tracked with *T*. *cruzi* CYP51 inhibition, akin to the known human CYP51 training set. The work described here has therefore enabled us to advance the first two series into hit-to-lead studies with a greater degree of confidence and oral activity with compounds from both series has subsequently been demonstrated in mouse models of Chagas disease.

**Fig 7:**
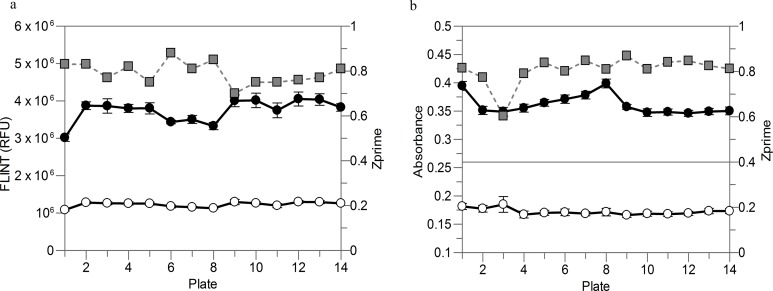
Trends of control 1 (closed circles, solvent only control, 0% inhibition), control 2 (open circles, reaction in absence of bactosomes, 100% inhibition) and Z’ (grey squares) of representative high-throughput screening runs of *T*. *cruzi* CYP51 FLINT (a) and cytochrome c reductase absorbance (b) assays in 384 well format. Error bars are the standard deviations of 16 replicates for each control and plate.

The 96 well plate based assay used in this analysis has now been further miniaturized and adapted to automated liquid dispensers and microplate readers for high-throughput screening in 384 well endpoint format. The *T*. *cruzi* CYP51 FLINT assay (Fig 7a) can be run in 20 μL and the cytochrome c reductase absorbance assay (Fig 7b) in 50 μL final volumes. This allows a throughput increase to up to more than 10,000 wells in a single experiment with Z’ > 0.7 and with a significant reduction in screening cost and time. Sensitivity to described inhibitors was maintained during assay miniaturization.
